# Herbal Medicine Classification: Policy Recommendations

**DOI:** 10.3389/fmed.2020.00031

**Published:** 2020-02-11

**Authors:** Azhar H. Alostad, Douglas T. Steinke, Ellen I. Schafheutle

**Affiliations:** Division of Pharmacy and Optometry, School of Health Sciences, Faculty of Biology, Medicine and Health, The University of Manchester, Manchester, United Kingdom

**Keywords:** policy reform, herbal medicine regulation, policy implementation, Kuwait, classifications

## Background

Herbal medicines (HMs) have been defined as “preparations manufactured industrially consisting of active ingredient(s) which is/are purely and naturally original, not chemically altered plant substance(s), and is/are responsible for the overall therapeutic effect of the product” (1). Due to a belief that as “natural” products, HMs are “safe” or “safer” than conventional medicines, consumers have turned to plant-derived preparations (2). However, similar to conventional medicines, HMs contain several chemical constituents, that are capable of producing pharmacological effects causing both mild and serious adverse effects, ranging from renal disfunction, liver toxicity, elevated blood pressure, and even death (3, 4). Safety issues of HMs may also arise when the product is contaminated with bacteria, yeast or mold, or is adulterated with orthodox medicines (4, 5).

The pre-marketing control of medicines in drug regulatory authorities (DRAs) helps to assure the safe use of all medicines, by performing appropriate quality, safety, and efficacy assessments prior to their marketing (6). To do so, DRAs require manufacturing companies to submit evidence of adherence to Good Manufacturing Practice (GMP) and quality control to demonstrate the product's quality, and evidence of toxicity screening to prove the product's safety. The research protocols, standards and methods required for the evaluation of HMs' efficacy are more complex and difficult to obtain (7). Unlike conventional medicines, a single HM may contain hundreds of natural constituents, and a mixed HM may contain several times more that number. This poses standardization, ethical and financial strains to perform clinical trials for HMs, limiting the ability of manufacturing companies to fulfill the requirement of clinical efficacy (8).

The occurrence of high-profile safety concerns of HMs, coupled with the difficulty to demonstrate clinical efficacy, mandated that DRAs have regulatory evaluation measures in place to ensure the safe use and availability of HMs in their market (9). However, in some countries, HMs are not regarded as medicines but as foods or dietary supplements, as such requiring less rigorous regulatory assessment than medicines (3). This global regulatory inconsistency on how countries define and classify HMs have an effect on small countries such as Kuwait, which lacks the capacity to manufacture its own HMs, and therefore imports all of its HMs from other countries. The issue in the Kuwaiti DRA structure is that a clear classification and definition of what is considered a HM for registration does not exist. Therefore, the classification of the product in the country of origin guides how the product is dealt with prior to marketing. This causes inconsistency in how products are assessed in the Kuwaiti DRA, with some HMs being assessed in the Herbal Department but others in other departments with less stringent requirements (3). In order to allow a standardized approach for evaluating the quality, safety and efficacy of imported HMs into Kuwait, and ensure public safety, it is essential to develop a clear HM definition and classification procedure in the Kuwaiti DRA structure and make recommendations for its implementation (10).

To inform such a proposal, the authors conducted a four step research study using Anderson's policy framework, which emphasizes using reliable evidence to inform future policy design and implementation (11). The first study consisted of a comparison of five established HMs registration systems that are either exporters of HMs into Kuwait [Germany, United Kingdom (UK), United States (US)], or countries that are culturally, financially and geographically similar to Kuwait [Bahrain and United Arab Emirates (UAE)] (1). The second study consisted of a review of relevant implementation literature (Alostad et al., under review). The third and fourth studies consisted of a qualitative exploration of an established system (Bahrain), which similarly to Kuwait, relies on registering HMs manufactured elsewhere, followed by an investigation of Kuwait's system and readiness for implementation (12). Using the findings from the above studies, the aim of this article is to provide a description and justification of the proposed definition, classification policy, and the plan to implement it, generated from the four studies.

## The Recommendations

### Proposed Definition and Classification

**Figure 1:**
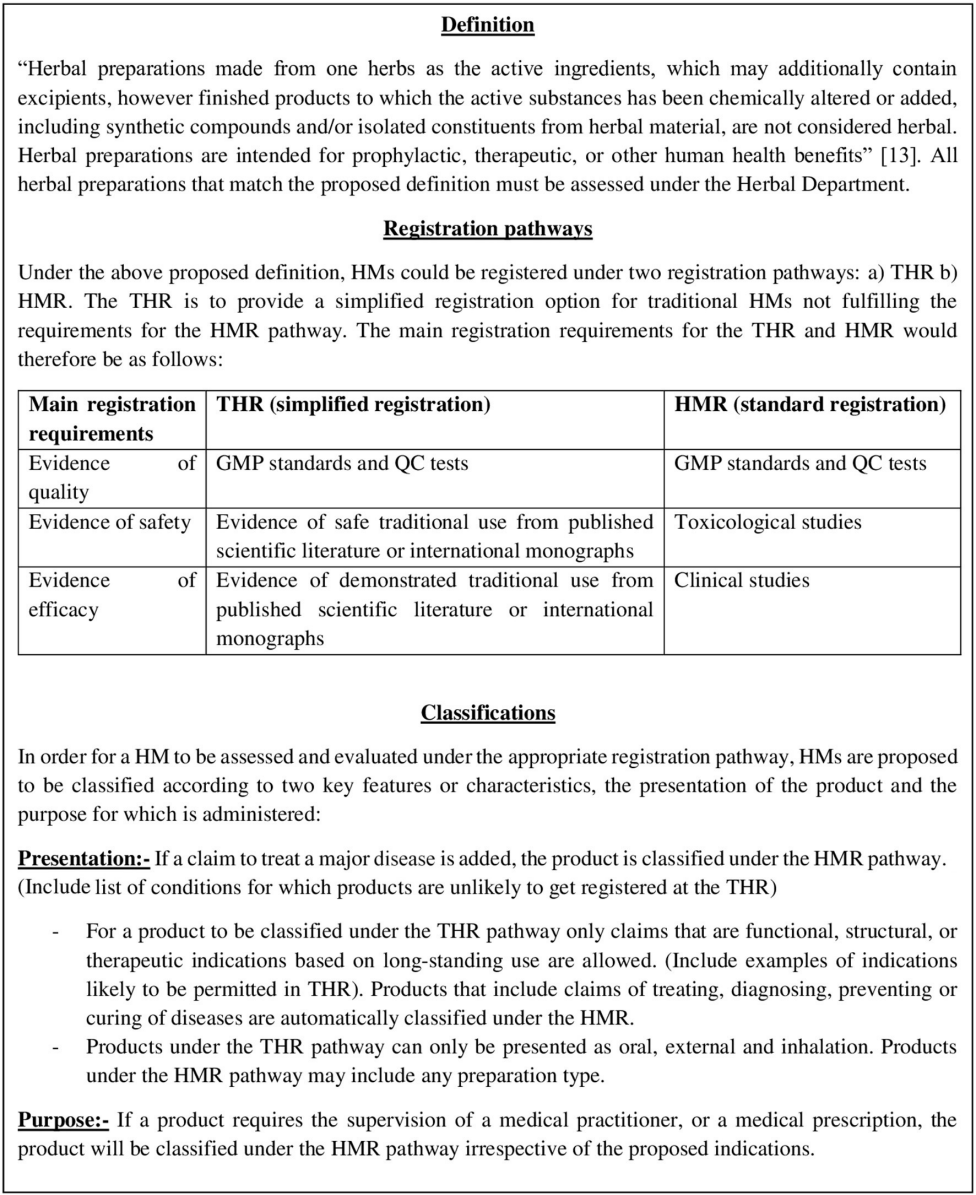
Preview of the proposed herbal medicine classification policy.

Findings from comparing the HM registration laws in five countries (1) revealed that all comparative authorities state in their HMs definitions that the product must consist of plant materials, with Germany, UK (under European Union Directive 2004/24/EC), Bahrain and UAE having the highest similarity in defining a HM. These four countries' definitions are consistent with the recommended World Health Organization (WHO) definition of an “authorized industrially manufactured therapeutic herbal product for registration” (13). To promote international harmonization, the WHO definition (Figure 1) can be considered for Kuwait, which has already been adopted and adapted in many countries including importing countries in Africa (14) and the Middle-East (15, 16).

The five-country comparison study (1) also showed that the US market HMs as “dietary supplements,” which does not require any pre-marketing evaluation. In the US, the regulation for dietary supplements is a reactive rather than proactive approach, whereby these products can be placed in the market with no quality, safety, or efficacy evaluation unless a safety incident has occurred or a case can be made that a particular product poses a specific danger to the public (3). Evidence already exists that classification of HMs as dietary supplements has resulted in serious and growing public health problems (17). A safer and more considered approach in registering HMs was revealed in the UK, Germany, UAE, and Bahrain, which all offer a simplified registration called the “traditional herbal registration (THR)” based on the product's presentation and purpose (Figure 1). In line with the THR scheme, in addition to performing quality and safety checks, “plausible efficacy” based on evidence of long-standing use and experience is sufficient to prove the product's efficacy. A study that evaluated the impact of the THR scheme in the UK prior to and after its implementation revealed that an increase in the quality of information provided with HMs was achieved (18). As the THR scheme is associated with appropriate pre-marketing evaluation, and more complete information than the dietary supplements classification, it is proposed that in addition to the choice of applying for a product license of the type needed to manufacture “conventional” products and providing evidence of clinical efficacy, the THR scheme should be implemented in the Kuwaiti DRA (Figure 1).

### Proposed Implementation Plan

Having recommended a definition and classification policy for the Kuwaiti DRA, it is essential to inform how these recommendations are to be implemented. In exploring the HM classification policy implementation process in Bahrain (including implementation facilitators and barriers) and implementation readiness in Kuwait (12), 10 recommendations are proposed to strategically plan potential implementation. These recommendations are consistent with the medicine policy implementation literature (19), which emphasizes the importance of management support and leadership, employees' involvement in the policymaking process, promoting a culture of appreciation and teamwork, and allocation of human, financial, and technical resources. The recommendations are as follows:

1. Adoption of a legally binding HM definition and classification guideline, with an additional route of evaluation and registration for HMs which are not classified as complete medicines [e.g., the Bahraini Pharmaceutical Classification Guideline (12)]. Furthermore, to avoid inconsistency in the review process, HMs matching the proposed definition must be assessed in one department (the Herbal Department).
2. To ensure independence and compliance, classification decisions should be made by two employees, with wider discussion where discrepancies are encountered.
3. To achieve good employee buy-in, understanding and adoption, management should involve them in detailed development and implementation of the policy.
4. Resource needs must be assessed prior to policy implementation, including: (a) financial resources; laboratory instruments for analyzing HMs, employee salaries, costs of training etc., (b) human resources; sufficient experienced staff with good knowledge about HMs, (c) specialized training in HMs and continuing professional development for employees, (d) access to international HMs references such as the Herbal Medicines Compendium, European Pharmacopoeia, Japanese Pharmacopoeia, and British Pharmacopoeia.
5. When the final policy has been approved, and in the interest of transparency, it should be published on the Kuwaiti DRA official website. Manufacturers should then be informed regarding content and implementation.
6. A transitional period should be given for traditional HMs already on the market, allowing sufficient time for all stakeholders, including manufacturers and agents, to adapt to the new requirements.
7. Promote leadership by identifying advocates that could facilitate and enhance acceptance of the new policy in a positive working environment.
8. Identify appropriate and independent monitoring measures (internal audit) by a separate department to ensure that the employees' performance meets the authority's demands and expectations. The audits can include obtaining employees' and manufacturers' feedback, inspections to monitor classification consistency and compliance through observing employees' performance and tracking of HMs applications.
9. Conduct a review of the policy (e.g., every 5 years) which could be achieved by considering to engage with external HM regulatory experts to ensure that the policy is updated according to international laws.
10. A future recommendation is the possibility of separating the DRA from the Ministry of Health to become a separate juristic body, thus ensuring decisions are guided by interests of public health and safety, and expediting decisions.

## Conclusions

Based on evidence and empirical research, an outline of a policy specifically for HMs classification was generated, in which by implementing it, a registration consistency, and an increasing safety level can be expected. Practical implementation steps were proposed to facilitate the transfer of the new policy. Although the proposed policy and implementation roadmap were generated for Kuwait, the policy can be adopted to promote international harmonization of HMs registration, and the implementation roadmap can be tailored for other countries that do not have such policy implemented. Countries in the Arab World may benefit from coordinating their definitions and registration systems for HMs, such that acceptance and registration of a particular HM in one Arab World country would lead to mutual recognition of this in all other countries. This could allow accelerated and more efficient registration for HMs for importing countries, but further studies would be required to evaluate such a procedure.

## Author Contributions

AA: writing of manuscript. DS and ES: revision of manuscript. ES: providing significant input. All authors reviewed the manuscript and agreed with the decision to submit for publication.

### Conflict of Interest

This is an independent research undertaken as part of a Ph.D., and the authors were not asked nor commissioned to inform a herbal medicine registration system for the Kuwaiti drug regulatory authority (DRA). However, it is projected that the recommendations listed in this manuscript will be proposed to the Kuwaiti DRA for consideration.

## Abbreviations

DRA: Drug Regulatory Authority
HM: Herbal Medicine
THR: Traditional Herbal Registration
UAE: United Arab Emirates
US: United States
WHO: World Health Organization.
